# Single-Nucleotide Polymorphism–Based Genetic Risk Score and Patient Age at Prostate Cancer Diagnosis

**DOI:** 10.1001/jamanetworkopen.2019.18145

**Published:** 2019-12-27

**Authors:** Rong Na, Craig Labbate, Hongjie Yu, Zhuqing Shi, Richard J. Fantus, Chi-Hsiung Wang, Gerald L. Andriole, William B. Isaacs, S. Lilly Zheng, Brian T. Helfand, Jianfeng Xu

**Affiliations:** 1 Program for Personalized Cancer Care, NorthShore University HealthSystem, Evanston, Illinois; 2 Huashan Hospital, Fudan Institute of Urology, Fudan University, Shanghai, China; 3 Ruijin Hospital, Department of Urology, Shanghai Jiao Tong University School of Medicine, Shanghai, China; 4 Section of Urology, University of Chicago Medicine, Chicago, Illinois; 5 Division of Urologic Surgery, Department of Surgery, Washington University School of Medicine in St Louis, St Louis, Missouri; 6 James Buchanan Brady Urological Institute, Department of Urology, Johns Hopkins University School of Medicine, Baltimore, Maryland; 7 Sidney Kimmel Comprehensive Cancer Center, Johns Hopkins University School of Medicine, Baltimore, Maryland

## Abstract

**Importance:**

Few studies have evaluated the association between a single-nucleotide polymorphism–based genetic risk score (GRS) and patient age at prostate cancer (PCa) diagnosis.

**Objectives:**

To test the association between a GRS and patient age at PCa diagnosis and to compare the performance of a GRS with that of family history (FH) in PCa risk stratification.

**Design, Setting, and Participants:**

A cohort study of 3225 white men was conducted as a secondary analysis of the Reduction by Dutasteride of Prostate Cancer Events (REDUCE) chemoprevention trial, a 4-year, randomized, double-blind, placebo-controlled multicenter study conducted from March 2003 to April 2009 to evaluate the safety and efficacy of dutasteride in reducing PCa events. Participants were confirmed to be cancer free by prostate biopsy (6-12 cores) within 6 months prior to the study and underwent 10 core biopsies every 2 years per protocol. The dates for performing data analysis were from July 2016 to October 2019.

**Interventions:**

A well-established, population-standardized GRS was calculated for each participant based on 110 known PCa risk–associated single-nucleotide polymorphisms, which is a relative risk compared with the general population. Men were classified into 3 GRS risk groups based on predetermined cutoff values: low (<0.50), average (0.50-1.49), and high (≥1.50).

**Main Outcomes and Measures:**

Prostate cancer diagnosis–free survival among men of different risk groups.

**Results:**

Among 3225 men (median age, 63 years [interquartile range, 58-67 years]) in the study, 683 (21%) were classified as low risk, 1937 (60%) as average risk, and 605 (19%) as high risk based on GRS alone. In comparison, 2789 (86%) were classified as low or average risk and 436 (14%) as high risk based on FH alone. Men in higher GRS risk groups had a PCa diagnosis–free survival rate that was worse than that of those in the lower GRS risk group (χ^2^ = 53.3; *P* < .001 for trend) and in participants with a negative FH of PCa (χ^2^ = 45.5; *P* < .001 for trend). Combining GRS and FH further stratified overall genetic risk, indicating that 957 men (30%) were at high genetic risk (either high GRS or positive FH), 1667 men (52%) were at average genetic risk (average GRS and negative FH), and 601 men (19%) were at low genetic risk (low GRS and negative FH). The median PCa diagnosis–free survival was 74 years (95% CI, 73-75 years) for men at high genetic risk, 77 years (95% CI, 75 to >80 years) for men at average genetic risk, and more than 80 years (95% CI, >80 to >80 years) for men at low genetic risk. In contrast, the median PCa diagnosis–free survival was 73 years (95% CI, 71-76 years) for men with a positive FH and 77 years (95% CI, 76-79 years) for men with a negative FH.

**Conclusions and Relevance:**

This study suggests that a GRS is significantly associated with patient age at PCa diagnosis. Combining FH and GRS may better stratify inherited risk than FH alone for developing personalized PCa screening strategies.

## Key Points

**Question:**

Is family history sufficient to identify men at high risk of prostate cancer?

**Findings:**

In a cohort study of 3225 men, family history alone identified 14% at high risk of prostate cancer and 86% at low or average risk; men with a positive family history of prostate cancer had a median diagnosis-free survival of 73 years, while men with a negative family history had a median diagnosis-free survival of 77 years. A combination of family history and single-nucleotide polymorphism–based genetic risk score, however, identified 30% of men at high genetic risk of prostate cancer, 52% of men at average genetic risk, and 19% of men at low genetic risk; the median diagnosis-free survival was 74 years for men at high genetic risk, 77 years for men at average genetic risk, and more than 80 years for men at low genetic risk.

**Meaning:**

Combining family history and genetic risk score can better stratify inherited risk to develop personalized prostate cancer screening strategies.

## Teaser

This cohort study examines the association between a single-nucleotide polymorphism (SNP)–based genetic risk score and patient age at prostate cancer diagnosis and compares the performance of the genetic risk score with that of family history in prostate cancer risk stratification.

## Introduction

Population-based prostate-specific antigen (PSA) screening for prostate cancer (PCa) has been subject to intense scrutiny owing to the potential harms of overdiagnosis and overtreatment.^1^ Despite data supporting a mortality benefit from population screening, it remains unclear which patient groups have the most favorable risk-benefit ratio from screening.^2^ Currently, all professional guidelines recommend that age, family history (FH), and race/ethnicity be considered to identify subsets of men who may benefit the most from PSA screening.^3,4,5,6^ The US Preventive Services Task Force recommends a discussion about the benefits and harms of PSA screening for men aged 55 to 69 years and states that FH and race/ethnicity be considered in determining whether earlier PSA screening is appropriate for individual men.^3^

Both FH and race/ethnicity are used to measure the inherited risk for PCa, a disease with strong heritability (*h*^2^ = 0.57).^7^ However, FH and race/ethnicity are indirect measurements of inherited risk, which can also be associated with environmental exposures. With advances in DNA sequencing and genotyping technologies and the identification of specific PCa susceptibility genes and variants,^8,9,10^ it is now feasible to also include direct DNA measurements as part of inherited risk assessment.

In the last decade, more than 170 PCa risk–associated single-nucleotide polymorphisms (SNPs) have been identified through multiple large genome-wide association studies (GWASs).^10^ Despite small individual associations of these SNPs with PCa risk, they have a stronger cumulative association.^11^ Several polygenic risk score methods have been used for measuring the cumulative association of SNPs.^12^ To date, all published studies, including those from large case-control studies,^10,13^ retrospective analysis of prospective studies,^14,15^ prostate biopsy cohorts,^16,17^ and prospective studies,^18^ consistently demonstrate associations of polygenic risk scores with PCa risk. These results provide a basis for a polygenic risk score to be incorporated in the assessment of inherited risk for PSA screening.

However, few studies of polygenic risk scores have evaluated their association with patient age at diagnosis of PCa, a critical piece of evidence that will not only strengthen their association with PCa risk but, more importantly, provide direct evidence for their use in determining patient age for PSA screening. Seibert and colleagues^19^ identified 54 SNPs that were associated with patient age at diagnosis of PCa in the PRACTICAL (Prostate Cancer Association Group to Investigate Cancer Associated Alterations in the Genome) consortium and then validated the association of a polygenic hazard score derived from these 54 SNPs with patient age at diagnosis in the Protect (Prostate Testing for Cancer and Treatment) study.^20^

In this study, we tested the association between polygenic risk using a genetic risk score (GRS) and patient age at PCa diagnosis in a randomized clinical trial population. The GRS is an odds ratio (OR)–weighted and population-standardized score derived from well-established PCa risk–associated SNPs, and this method has been previously described.^12^

## Methods

### Study Population

The Reduction by Dutasteride of Prostate Cancer Events (REDUCE) chemoprevention trial was a 4-year, randomized, double-blind, placebo-controlled study conducted from March 2003 to April 2009 to evaluate the safety and efficacy of dutasteride in reducing PCa events.^21^ Patients were between the ages of 50 and 75 years at enrollment, with a PSA level between 2.5 and 10 ng/mL (to convert to micrograms per liter, multiply by 1.0). Participants were confirmed to be cancer free by prostate biopsy (6-12 cores) within 6 months prior to the study and underwent 10 core transrectal ultrasonography-guided biopsies every 2 years per protocol. Participants who did not have a diagnosis of PCa at completion of the study were censored. High-grade PCa was defined as a Gleason grade of 3 + 4 or greater. A positive FH was defined as any first-degree relative with PCa. The present genetic study was limited to a cohort of white men who provided written informed consent for genetic research (3225 of 6729 men in the final analysis of the REDUCE trial). The collection of DNA data was performed prospectively prior to randomization. The institutional review boards at all participating institutions had approved of the original study. The current retrospective genetic analysis received approval from the Wake Forest School of Medicine Institutional Review Board. We followed the Strengthening the Reporting of Observational Studies in Epidemiology (STROBE) reporting guideline. The dates for performing data analysis were from July 2016 to October 2019.

### PCa Risk–Associated SNP Genotyping

We searched PubMed for PCa GWASs published prior to July 1, 2018, and identified independent PCa risk–associated SNPs using the following standard criteria: (1) discovered from GWASs of white men, with at least 1000 cases and 1000 controls in the first stage; (2) confirmed in additional stages with combined *P* < 5 × 10^−8^; and (3) independent, linkage disequilibrium measurement (*r*^2^ < 0.2) between any pair of SNPs. Of the 174 SNPs that met these criteria, 110 PCa risk–associated SNPs were available directly and indirectly (with imputation using the combined data of the 1000 Genomes Project^22^ and HapMap3^23^ data) from the genotyping data of the HumanOmniExpress BeadChip (Illumina Inc). Details regarding these SNPs, including whether they were directly genotyped or imputed, are described in the eTable in the Supplement, and detailed methods of genotyping, imputation, and quality control have been previously described.^24^

### Calculation of GRS

An OR-weighted and population-standardized GRS was computed for each participant based on the 110 PCa risk–associated SNPs.^12^ In brief, a GRS was calculated by multiplying the per-allele OR for each SNP and normalizing the risk by the mean risk expected in the population:
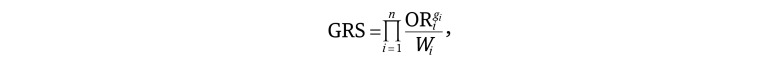


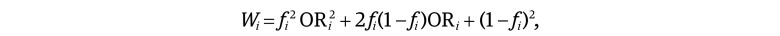
where *W* stands for population, *g*_i_ stands for the genotype of SNP *i* for an individual (0, 1, or 2 risk alleles), OR*_i_* stands for the OR of SNP *i*, and *f_i_* stands for the risk allele frequency of SNP *i*. Allelic ORs obtained from the external studies and allele frequencies in the gnomAD (Non-Finnish European population) were used in the calculation. The gnomAD included 55 860 Non-Finnish European individuals, which to our knowledge is the largest sequenced population of broad European descent.

Because GRS is population standardized, its mean is expected to be 1.0 and its values can be interpreted as relative risk to the general population. In this study, participants were classified into 3 risk groups prior to the analysis based on GRS values: low (<0.50), average (0.50-1.49), and high risk (≥1.50). The cutoff value of 1.5 is determined benchmarked to the PCa risk conferred by a positive FH. A positive FH is associated with 1.5-fold increased risk for PCa from multiple prospective studies.^25^

### Statistical Analysis

For univariate analysis, differences of quantitative variables among groups were tested using analysis of variance, and differences of qualitative variables among groups were tested using the χ^2^ test. The detection rates of PCa and high-grade PCa among groups were tested using the χ^2^ test (the trend test for groups with increasing risk). As PCa-free survival on an age scale is likely to be left-truncated owing to the fact that patients with a prior PCa diagnosis were not included in the REDUCE trial, we used a Kaplan-Meier survival analysis package in R, version 3.5.2 (R Project for Statistical Computing) with a left-truncated method to assess the association between GRS and patient age at PCa diagnosis. Multivariate analyses were performed to test for the independent effect of factors associated with PCa diagnosis using a linear regression model (for patient age at diagnosis), a logistic regression model (for diagnosis of PCa and high-grade PCa), and a Cox proportional hazards regression model (for PCa diagnosis–free survival time). Statistical analyses were performed using R version 3.5.2, and a 2-tailed *P* < .05 was considered statistically significant.

## Results

During the 4-year follow-up of 3225 participants included in the genetic subcohort of the REDUCE trial (1644 participants from the placebo group and 1581 participants from the dutasteride group), a diagnosis of PCa was made for 714 participants (22%), and a diagnosis of high-grade PCa was made for 237 participants (7%). Based on GRSs alone, 683 participants (21%) in the study were classified as low risk (<0.5), 1937 (60%) as average risk (0.50-1.49), and 605 (19%) as high risk (≥1.50). There was no significant difference in age, FH, and total PSA levels among GRS groups (Table 1).

**Table 1.** Clinical and Demographic Information of Study Participants at Baseline

Risk Group	Men, No. (%) (N = 3225)	Age, Median (IQR), y	Family History, No. (%)	PSA Level, Median (IQR), ng/mL
GRS				
<0.50	683 (21)	63 (59-68)	82 (12)	5.6 (4.3-7.1)
0.50-1.49	1937 (60)	63 (58-67)	270 (14)	5.7 (4.4-7.3)
≥1.50	605 (19)	63 (58-68)	84 (14)	5.7 (4.4-7.3)
*P* value	NA	.14	.23	.91

Abbreviations: GRS, genetic risk score; IQR, interquartile range; NA, not applicable; PSA, prostate-specific antigen.

SI conversion factor: To convert PSA to micrograms per liter, multiply by 1.0.

The GRS risk groups were significantly associated with risk of PCa diagnosis during the 4-year follow-up (Table 2). The detection rate of PCa was 14% (95 of 683) for participants in the low GRS risk group, 22% (426 of 1937) for participants in the average GRS risk group, and 32% (193 of 605) for participants in the high GRS risk group (χ^2^ = 60.3; *P* < .001 for trend). Family history was also significantly associated with PCa diagnosis; the detection rate of PCa was 27% (116 of 436) for men with a positive FH, slightly higher than for men with a negative FH (598 of 2789 [21%]) (χ^2^ = 5.83; *P* = .02). Fourteen percent of participants (436 of 3225) had a positive FH, and 86% (2789 of 3225) had a negative FH.

**Table 2.** Detection Rate of Prostate Cancer by GRS and FH

Risk Group	Participants, No. (%)	Patients With PCa, No. (%)	
		All PCa	High-Grade PCa
**All Participants (N = 3225)**			
GRS			
<0.50	683 (21)	95 (14)	34 (5)
0.50-1.49	1937 (60)	426 (22)	143 (7)
≥1.50	605 (19)	193 (32)	60 (10)
*P* value for trend	NA	<2.2 × 10^−16^	2.4 × 10^−4^
**All Participants (N = 3225)**			
Negative FH	2789 (86)	598 (21)	198 (7)
Positive FH	436 (14)	116 (27)	39 (9)
*P* value	NA	.02	.17
**Participants With Positive FH (n = 436)**			
GRS			
<0.50	82 (19)	15 (18)	6 (7)
0.50-1.49	270 (62)	70 (26)	24 (9)
≥1.50	84 (19)	31 (37)	9 (11)
*P* value for trend	NA	6.01 × 10^−5^	.40
**Participants With Negative FH (n = 2789)**			
GRS			
<0.50	601 (22)	80 (13)	28 (5)
0.50-1.49	1667 (60)	356 (21)	119 (7)
≥1.50	521 (19)	162 (31)	51 (10)
*P* value for trend	NA	<2.2 × 10^−16^	3.12 × 10^−4^
**All Participants (N = 3225)**			
Negative FH and GRS <0.50	601 (19)	80 (13)	28 (4)
Negative FH and GRS 0.50-1.49	1667 (52)	356 (21)	119 (7)
Positive FH or GRS ≥1.50	957 (30)	278 (29)	90 (9)
*P* value for trend	NA	1.76 × 10^−13^	4.2 × 10^−4^

Abbreviations: FH, family history; GRS, genetic risk score; NA, not applicable; PCa, prostate cancer.

**Figure 1.**
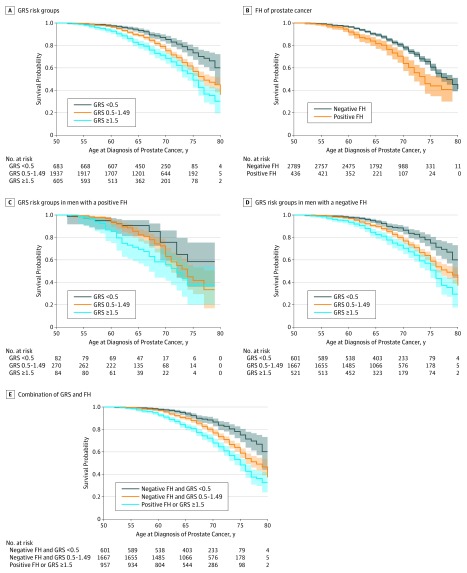
Prostate Cancer Diagnosis–Free Survival Curves Based on 4-Year Follow-up of 3225 Participants in the REDUCE (Reduction by Dutasteride of Prostate Cancer Events) Trial A, Genetic risk score (GRS) risk groups. B, Family history (FH). C, The GRS risk groups for men with a positive FH of prostate cancer. D, The GRS risk groups for men with a negative FH of prostate cancer. E, Combination of GRS and FH of prostate cancer. Shaded areas indicate 95% CIs.

Prostate cancer diagnosis–free survival was estimated for participants based on GRS risk groups and FH (Table 3). The PCa diagnosis–free survival was significantly worse for participants of higher GRS risk groups (χ^2^ = 53.3; log-rank *P* < .001 for trend; Figure 1A). By the age of 75 years, the probability of PCa diagnosis–free survival was 0.72 (95% CI, 0.67-0.78) for men in the low GRS risk group, 0.56 (95% CI, 0.52-0.60) for men in the average risk group, and 0.47 (95% CI, 0.41-0.53) for men in the high GRS risk group. The median PCa diagnosis-free survival was more than 80 years (95% CI, >80 to >80 years) for men in the low GRS risk group, 76 years (95% CI, 74 to >80 years) for men in the average GRS risk group, and 74 years (95% CI, 72-78 years) for men in the high GRS risk group. Similarly, the PCa diagnosis–free survival was worse for men with a positive FH (χ^2^ = 21.9; log-rank *P* < .001; Figure 1B). The probability of PCa diagnosis-free survival by the age of 75 years was 0.59 (95% CI, 0.56-0.62) for men with a negative FH and 0.43 (95% CI, 0.35-0.52) for men with a positive FH. The median PCa diagnosis–free survival was 77 years (95% CI, 76-79 years) for men with a negative FH and 73 years (95% CI, 71-76 years) for men with a positive FH. More importantly, GRS risk groups remained a significant factor associated with PCa diagnosis–free survival for men with a positive FH (χ^2^ = 7.2; log-rank *P* = .03 for trend; Figure 1C) and a negative FH (χ^2^ = 45.5; *P* < .001 for trend; Figure 1D).

**Table 3.** Prostate Cancer Disease–Free Survival by GRS and FH

Risk Group	Men, No. (%)	Cumulative Probability (95% CI) by 75 y of Age		Age (95% CI) at 50% Survival, y	
		All PCa	High-Grade PCa	All PCa	High-Grade PCa
**All Participants (N = 3225)**					
GRS					
<0.50	683 (21)	0.72 (0.67-0.78)	0.89 (0.84-0.93)	>80 (>80 to >80)^a^	>80 (>80 to >80)^a^
0.50-1.49	1937 (60)	0.56 (0.52-0.60)	0.80 (0.76-0.84)	76 (74 to >80)	>80 (>80 to >80)^a^
≥1.50	605 (19)	0.47 (0.41-0.53)	0.78 (0.72-0.85)	74 (72 to 78)	>80 (>80 to >80)^a^
**All Participants (N = 3225)**					
Negative FH	2789 (86)	0.59 (0.56-0.62)	0.83 (0.80-0.86)	77 (76 to 79)	>80 (>80 to >80)^a^
Positive FH	436 (14)	0.43 (0.35-0.52)	0.74 (0.65-0.84)	73 (71 to 76)	>80 (>80 to >80)^a^
**Participants With Positive FH (n = 436)**					
GRS					
<0.50	82 (19)	0.57 (0.41-0.79)	0.85 (0.75-0.97)	>80 (>80 to >80)^a^	>80 (>80 to >80)^a^
0.50-1.49	270 (62)	0.41 (0.31-0.54)	0.72 (0.60-0.87)	73 (71 to 78)	>80 (>80 to >80)^a^
≥1.50	84 (19)	0.35 (0.22-0.56)	0.69 (0.52-0.92)	70 (66 to 74)	>80 (>80 to >80)^a^
**Participants With Negative FH (n = 2789)**					
GRS					
<0.50	601 (22)	0.74 (0.68-0.80)	0.90 (0.86-0.95)	>80 (>80 to >80)^a^	>80 (>80 to >80)^a^
0.50-1.49	1667 (60)	0.58 (0.54-0.62)	0.81 (0.77-0.85)	77 (75 to >80)	>80 (>80 to >80)^a^
≥1.50	521 (19)	0.48 (0.42-0.55)	0.79 (0.73-0.86)	74 (73 to 77)	>80 (>80 to >80)^a^
**All Participants (N = 3225)**					
Negative FH and GRS <0.50	601 (19)	0.74 (0.68-0.80)	0.90 (0.84-0.94)	>80 (>80 to >80)^a^	>80 (>80 to >80)^a^
Negative FH and GRS 0.50-1.49	1667 (52)	0.58 (0.54-0.62)	0.81 (0.77-0.85)	77 (75 to >80)	>80 (>80 to >80)^a^
Positive FH or GRS ≥1.50	957 (30)	0.46 (0.41-0.52)	0.76 (0.72-0.83)	74 (73 to 75)	>80 (>80 to >80)^a^

Abbreviations: FH, family history; GRS, genetic risk score; PCa, prostate cancer.

^a^ The 95% CIs are the same as the age itself because more than 80 years was the upper limit.

When GRS and FH were used jointly to stratify overall genetic risk, 601 of 3225 men (19%) were classified as low genetic risk (low GRS and negative FH), 1667 of 3225 men (52%) as average genetic risk (average GRS and negative FH), and 957 of 3225 (30%) of men as high genetic risk (either high GRS or positive FH). Prostate cancer diagnosis–free survival was significantly worse for participants of higher GRS risk groups (χ^2^ = 63.0; log-rank *P* < .001; Figure 1E). The probability of PCa diagnosis-free survival by the age of 75 years was 0.74 (95% CI, 0.68-0.80) for men at low genetic risk, 0.58 (95% CI, 0.54-0.62) for men at average genetic risk, and 0.46 (95% CI, 0.41-0.52) for men at high genetic risk. The median PCa diagnosis–free survival was more than 80 years (95% CI, >80 to >80 years) for men at low genetic risk, 77 years (95% CI, 75 to >80 years) in men at average genetic risk, and 74 years (95% CI, 73-75 years) for men at high genetic risk (Table 3).

A multivariate Cox proportional hazards regression analysis including GRS (continuous variable), FH, age, and baseline PSA level showed GRS (β = 0.28; *P* < .001) and FH (β = 0.516; *P* < .001) to be independent factors associated with PCa diagnosis–free survival. In addition, there was no interaction between GRS and FH (χ^2^ = 0.19; *P* = .91). Results were similar when the analyses were performed separately in groups that received placebo or dutasteride (eFigure 1 and eFigure 2 in the Supplement).

**Figure 2.**
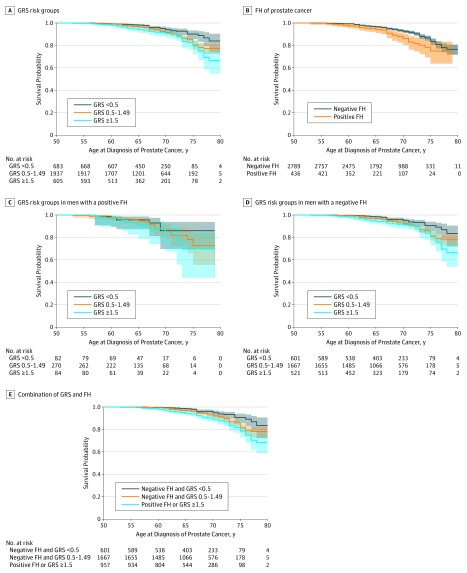
High-Grade Prostate Cancer Diagnosis–Free Survival Curves Based on 4-Year Follow-up of 3225 Participants in the REDUCE (Reduction by Dutasteride of Prostate Cancer Events) Trial A, Genetic risk score (GRS) risk groups. B, Family history (FH). C, The GRS risk groups for men with a positive FH of prostate cancer. D, The GRS risk groups for men with a negative FH of prostate cancer. E, Combination of GRS and FH of prostate cancer. Shaded areas indicate 95% CIs.

Similar analyses were performed for high-grade PCa. Genetic risk score group was significantly associated with risk of high-grade PCa (χ^2^ = 11.5; *P* < .001 for trend). In contrast, FH was not significantly associated with diagnosis of high-grade PCa (χ^2^ = 1.89; *P* = .17) (Table 2). The high-grade PCa diagnosis–free survival was significantly worse for men in higher GRS risk groups (χ^2^ = 12.8; log-rank *P* = .002 for trend; Figure 2A) and with a positive FH (χ^2^ = 8.8; log-rank *P* = .003 for trend; Figure 2B). In stratified analysis based on FH, higher GRS risk groups were not significantly associated with high-grade PCa diagnosis–free survival for men with a positive FH (χ^2^ = 0.9; log-rank *P* = .64 for trend; Figure 2C), but they were significantly associated with high-grade PCa diagnosis–free survival for men without a FH (χ^2^ = 11.9; log-rank *P* = .003 for trend; Figure 2D) and for men with risk stratified based on a combination of GRS and FH (χ^2^ = 18.5; log-rank *P* < .001 for trend; Figure 2E).

## Discussion

To our knowledge, this is the first reported study assessing the association of a polygenic risk score derived from well-established risk-associated SNPs with patient age at PCa diagnosis. We found that higher GRSs were significantly associated with an earlier age at PCa diagnosis. Furthermore, the association of GRS with patient age at PCa diagnosis was independent of FH. As a result, a combination of GRS and FH offers a more informative tool for risk stratification than does FH alone, the current standard of care in the assessment of inherited risk.

Since the first report in 2008 of a strong cumulative association between 5 PCa risk–associated SNPs discovered from GWASs and PCa risk,^11^ many studies have consistently demonstrated a significant association between polygenic risk score and PCa risk.^13,14,15,16,17,18^ However, few studies evaluated its association with patient age at diagnosis of PCa. The only prior study of polygenic risk with patient age at PCa diagnosis showed a clear association, but it used a different method of targeting SNPs based on association with age.^19^ None of the SNPs used in that study overlapped with SNPs previously identified and validated in GWASs for general PCa risk.

The results from the present study add additional critical evidence to support the inclusion of GRS to FH for better risk stratification. Although FH is currently the criterion standard measurement of assessment of inherited risk and is incorporated into multiple guidelines (US Preventive Services Task Force, American Urological Association, European Association of Urology, and National Comprehensive Cancer Network),^3,4,5,6^ it has multiple pitfalls. Fundamentally, FH is an indirect measurement of risk based on family members who share only partial genetic information. Furthermore, a complete FH is often difficult to obtain owing to age, family communication, and survival status of male relatives. In addition, FH is insufficient to identify individuals with higher inherited risk and will miss more than 50% of high-risk men in the population. Adding the GRS to FH creates a more complete genetic risk assessment.

The lack of association between FH and GRS risk groups in the our study is consistent with other previous studies.^10,11,14,25^ The exact reason is unclear but is likely due to a combination of the multiple factors. First, FH reflects both genetic factors and shared household environmental factors. Second, the GRS captures only partial genetic information from a subset of known risk-associated SNPs. Other inherited factors such as rare high-penetrant genes (eg, *BRCA2*, *ATM*, and *HOXB13*) and other yet to be identified common risk-associated SNPs may contribute to FH.^25^ Third, accurate FH information is difficult to obtain.

This present study also differs from other studies using the genetic subcohort of the REDUCE trial in that they all focused on GRS and PCa risk. In 2012, Kader et al^14^ demonstrated the association of higher GRS with higher PCa detection rates during the 4-year follow-up. The GRS in that study was based on the first 33 PCa risk–associated SNPs discovered at that time. With more PCa risk–associated SNPs subsequently identified from GWASs, Chen et al^26^ assessed the degree and association of risk reclassification with the increasing number of SNPs used in the calculation of the GRS (from 17, 34, and 51 to 68 SNPs). Results showed that risk reclassification was minimal with additional newly identified SNPs and suggested that currently available PCa risk–associated SNPs are reliable for risk stratification despite potential for the discovery of additional SNPs. Most recently, using the REDUCE cohort, Yu et al^27^ assessed the reliability of GRSs using a calibration benchmark. Results showed that a wide range of GRSs (ie, estimated risks) from the current 110-SNP panel was corroborated by observed PCa (actual risk) during the 4-year follow-up, with a high calibration slope and low bias score.

The significant association of GRS and patient age at PCa diagnosis found in the study is unlikely owing to potential bias in study design. First, the GRS method is well established to measure the cumulative effect of SNPs and has been associated with risk of multiple cancers and cardiovascular disease.^10,12,28,29,30^ Second, the PCa risk–associated SNPs (ORs and allele frequencies) used in the GRS calculation were discovered and confirmed from other large study populations (eTable in the Supplement). Third, the OR and allele frequency used in the GRS calculation are also derived from external study populations. Fourth, the dose-response association of GRS risk groups with PCa diagnosis-free survival curves provides further support for its validity. Fifth, the GRS, as an objective measurement, is not susceptible to self-reporting bias or to observers’ bias (because the GRS is unknown to test participants and investigators, practically double-blinded). Furthermore, as a germline marker, the GRS always precedes any phenotypes (regardless of the study design, retrospective or prospective) and therefore avoids temporal ambiguity. However, caution should be exercised when interpreting the estimates of PCa-free survival at the age of 75 years because a substantial proportion of censored patients (2223 of 3225 [69%]) were censored prior to the age of 75 years without a PCa diagnosis.

### Limitations

Several limitations should be noted. First, only men of European ancestry were included owing to limited the minority participants available in this study (only 2.3% were black, 1.6% Asian, and 4.0% American Hispanic); therefore, these data are not yet generalizable to other races/ethnicities. Second, half the study population was exposed to dutasteride with a potential chemopreventative effect. We performed survival analysis separately in the groups with or without dutasteride exposure and obtained similar findings (eFigure 1 and eFigure 2 in the Supplement). Third, our study population was substantially older than the current recommended ages to begin screening for PCa. It is yet to be demonstrated whether the data presented in this study can be extrapolated to clinically important screening decisions in the fifth and sixth decades of life. Fourth, the REDUCE trial included men with a prior negative biopsy result (owing to a moderately elevated PSA level), and such a population might not be representative of the entire screening population (most people in the screening population would have a normal PSA level). Further study based on the screening population is necessary to provide more robust evidence before the clinical use of the GRS for personalized screening. Fifth, men who participated in this trial might be motivated in PCa prevention. They may pay more attention to their health conditions, dietary habits, and other factors, which can lead to potential biases.

## Conclusions

This study suggests that the GRS is significantly associated with PCa risk and patient age at PCa diagnosis, and the association is independent of FH. Combining FH and GRS can better stratify inherited risk than FH alone. Such an inherited risk stratification strategy will benefit not only men at high risk by recommending earlier and more frequent PCa screening but also men at low risk by recommending decreased or delayed PCa screening.

## Notes

Supplement. **eTable.** Known Prostate Cancer Risk–Associated SNPs Available in the REDUCE Study **eFigure 1.** PCa Diagnosis-Free Survival Curves Based on 4-Year Follow-up of 1644 Subjects in the Placebo Arm of the REDUCE Study and Stratified by GRS Risk Groups (A), Family History (B), GRS Risk Groups in Men With a Positive Family History (C), and GRS Risk Groups in Men With a Negative Family History (D) **eFigure 2.** PCa Diagnosis-Free Survival Curves Based on 4-Year Follow-up of 1581 Subjects in the Dutasteride Arm of the REDUCE Study and Stratified by GRS Risk Groups (A), Family History (B), GRS Risk Groups in Men With a Positive Family History (C), and GRS Risk Groups in Men With a Negative Family History (D)
