# Supplementary material for: Single-Nucleotide Polymorphism–Based Genetic Risk Score and Patient Age at Prostate Cancer Diagnosis
Source: JAMA Netw Open. 2019 Dec 27;2(12):e1918145. doi: 10.1001/jamanetworkopen.2019.18145 (PMC6991229; doi:10.1001/jamanetworkopen.2019.18145)
Supplement: Supplement. — eTable. Known Prostate Cancer Risk–Associated SNPs Available in the REDUCE Study eFigure 1. PCa Diagnosis-Free Survival Curves Based on 4-Year Follow-up of 1644 Subjects in the Placebo Arm of the REDUCE Study and Stratified by GRS Risk Groups (A), Family History (B), GRS Risk Groups in Men With a Positive Family History (C), and GRS Risk Groups in Men With a Negative Family History (D) eFigure 2. PCa Diagnosis-Free Survival Curves Based on 4-Year Follow-up of 1581 Subjects in the Dutasteride Arm of the REDUCE Study and Stratified by GRS Risk Groups (A), Family History (B), GRS Risk Groups in Men With a Positive Family History (C), and GRS Risk Groups in Men With a Negative Family History (D) [file jamanetwopen-2-e1918145-s001.pdf]

Supplementary Online Content

Na R, Labbate C, Yu H, et al. Single-nucleotide polymorphism–based genetic risk score and patient age at prostate cancer diagnosis. *JAMA Netw Open.* 2019;2(12):e1918145. doi:10.1001/jamanetworkopen.2019.18145

eTable. Known Prostate Cancer Risk–Associated SNPs Available in the REDUCE Study
eFigure 1. PCa Diagnosis-Free Survival Curves Based on 4-Year Follow-up of 1644 Subjects in the Placebo Arm of the REDUCE Study and Stratified by GRS Risk Groups (A), Family History (B), GRS Risk Groups in Men With a Positive Family History (C), and GRS Risk Groups in Men With a Negative Family History (D)
eFigure 2. PCa Diagnosis-Free Survival Curves Based on 4-Year Follow-up of 1581 Subjects in the Dutasteride Arm of the REDUCE Study and Stratified by GRS Risk Groups (A), Family History (B), GRS Risk Groups in Men With a Positive Family History (C), and GRS Risk Groups in Men With a Negative Family History (D)

This supplementary material has been provided by the authors to give readers additional information about their work.

© 2019 Na R et al. *JAMA Network Open.*

eTable. Known Prostate Cancer Risk–Associated SNPs Available in the REDUCE Study						
SNP	CHR	POS	RA	RAF*	Published OR	Method
rs636291	1	10556097	A	0.69	1.1	Genotype
rs17599629	1	150658287	G	0.21	1.07	Genotype
rs1218582	1	154834183	G	0.43	1.05	Impute
rs4245739	1	204518842	A	0.76	1.1	Genotype
rs62106670	2	8456992	T	0.4	1.05	Impute
rs11902236	2	10117868	T	0.27	1.07	Genotype
rs9287719	2	10710730	C	0.45	1.07	Impute
rs9306895	2	20678392	C	0.38	1.08	Impute
rs1465618	2	43553949	T	0.23	1.09	Impute
rs721048	2	63131731	A	0.17	1.1	Genotype
rs10187424	2	85794297	T	0.59	1.08	Genotype
rs11691517	2	111135518	T	0.75	1.07	Impute
rs12621278	2	173311553	A	0.94	1.27	Impute
rs34925593	2	173369818	C	0.5	1.05	Impute
rs2292884	2	238443226	G	0.23	1.06	Genotype
rs3771570	2	242382864	T	0.15	1.09	Impute
rs2660753	3	87110674	T	0.12	1.13	Genotype
rs7611694	3	113556777	A	0.57	1.09	Genotype
rs10934853	3	128038373	A	0.29	1.1	Genotype
rs6763931	3	141102833	A	0.43	1.04	Genotype
rs10936632	3	170130102	A	0.52	1.1	Impute
rs10009409	4	73855253	T	0.31	1.06	Genotype

© 2019 Na R et al. *JAMA Network Open.*

rs1894292	4	74349158	G	0.53	1.06	Impute
rs17021918	4	95562877	C	0.65	1.09	Impute
rs7679673	4	106061534	C	0.58	1.13	Genotype
rs2242652	5	1280028	G	0.8	1.17	Impute
rs12653946	5	1895829	T	0.42	1.08	Genotype
rs2121875	5	44365545	C	0.32	1.05	Genotype
rs6869841	5	172939426	T	0.21	1.07	Impute
rs4976790	5	178541913	T	0.11	1.08	Genotype
rs4713266	6	11219030	C	0.53	1.05	Impute
rs7767188	6	30073776	A	0.21	1.06	Impute
rs12665339	6	30633454	G	0.17	1.06	Genotype
rs130067	6	31118511	G	0.2	1.05	Genotype
rs3096702	6	32192331	A	0.36	1.06	Impute
rs9296068	6	33020917	T	0.65	1.05	Genotype
rs9469899	6	34825346	A	0.37	1.05	Impute
rs1983891	6	41536427	T	0.29	1.09	Impute
rs4711748	6	43726860	T	0.22	1.05	Impute
rs9443189	6	76495882	A	0.87	1.07	Impute
rs2273669	6	109285189	G	0.15	1.07	Genotype
rs339331	6	117210052	T	0.68	1.09	Genotype
rs1933488	6	153441079	A	0.56	1.08	Genotype
rs9364554	6	160833664	T	0.27	1.11	Genotype
rs12155172	7	20994491	A	0.24	1.1	Genotype
rs10486567	7	27976563	G	0.77	1.14	Impute
rs17621345	7	40835592	A	0.74	1.07	Genotype

© 2019 Na R et al. *JAMA Network Open.*

rs56232506	7	47437244	A	0.47	1.06	Impute
rs6465657	7	97816327	C	0.48	1.11	Genotype
rs1512268	8	23526463	T	0.43	1.14	Genotype
rs2928679	8	23581461	A	0.43	1.05	Genotype
rs11135910	8	25892142	T	0.15	1.08	Genotype
rs12543663	8	126912413	C	0.31	1.12	Impute
rs620861	8	127323427	G	0.64	1.15	Impute
rs7837688	8	127527114	T	0.11	1.43	Impute
rs10086908	8	128011937	T	0.71	1.13	Genotype
rs1016343	8	128093297	T	0.21	1.25	Genotype
rs13252298	8	128095156	A	0.7	1.11	Genotype
rs6983561	8	128106880	C	0.03	1.13	Genotype
rs16902094	8	128320346	G	0.16	1.2	Impute
rs6983267	8	128413305	G	0.51	1.22	Genotype
rs1048169	9	19055966	C	0.38	1.06	Impute
rs1182	9	129813780	A	0.22	1.06	Impute
rs10993994	10	51549496	T	0.37	1.23	Genotype
rs1935581	10	88435391	C	0.62	1.05	Genotype
rs3850699	10	104414221	A	0.68	1.07	Genotype
rs4962416	10	126696872	C	0.27	1.06	Genotype
rs1881502	11	1486281	T	0.2	1.06	Genotype
rs7127900	11	2212343	A	0.2	1.19	Genotype
rs61890184	11	7526355	A	0.12	1.07	Impute
rs2277283	11	62140967	C	0.32	1.06	Impute
rs10896449	11	68994667	G	0.48	1.15	Genotype

© 2019 Na R et al. *JAMA Network Open.*

rs11568818	11	102401661	T	0.57	1.08	Genotype
rs11214775	11	113807181	G	0.72	1.07	Genotype
rs878987	11	134396477	G	0.16	1.07	Impute
rs2066827	12	12718164	T	0.77	1.06	Genotype
rs10845938	12	14263983	G	0.58	1.06	Genotype
rs10875943	12	49676010	C	0.3	1.07	Genotype
rs902774	12	53273904	A	0.15	1.13	Genotype
rs7968403	12	64619043	T	0.65	1.06	Impute
rs1270884	12	114685571	A	0.49	1.07	Impute
rs7295014	12	132491402	G	0.33	1.05	Impute
rs1004030	14	22836439	T	0.58	1.05	Impute
rs8008270	14	53372330	C	0.81	1.09	Genotype
rs7141529	14	69126744	C	0.48	1.05	Genotype
rs8014671	14	71092256	G	0.6	1.05	Genotype
rs11863709	16	57620663	C	0.97	1.16	Impute
rs684232	17	618965	C	0.36	1.09	Genotype
rs28441558	17	7899799	C	0.06	1.16	Impute
rs11649743	17	36074979	G	0.82	1.13	Genotype
rs4430796	17	36098040	A	0.55	1.22	Genotype
rs11650494	17	47345186	A	0.07	1.1	Impute
rs2680708	17	58378758	G	0.6	1.05	Impute
rs1859962	17	69108753	G	0.49	1.17	Genotype
rs28607662	18	55563627	C	0.09	1.08	Impute
rs12956892	18	59079082	T	0.3	1.05	Impute
rs10460109	18	75324209	T	0.43	1.04	Impute

© 2019 Na R et al. *JAMA Network Open.*

rs7241993	18	76773973	C	0.7	1.08	Genotype
rs11666569	19	17103262	C	0.72	1.05	Impute
rs8102476	19	38735613	C	0.54	1.09	Genotype
rs11672691	19	41985587	G	0.74	1.1	Genotype
rs61088131	19	42196794	T	0.84	1.06	Impute
rs2735839	19	51364623	G	0.87	1.18	Genotype
rs12480328	20	49527922	T	0.93	1.11	Impute
rs2427345	20	61015611	C	0.63	1.05	Genotype
rs6062509	20	62362563	T	0.7	1.08	Impute
rs1041449	21	42901421	G	0.44	1.05	Impute
rs5759167	22	43500212	G	0.5	1.15	Genotype
rs2405942	X	9814135	A	0.78	1.05	Genotype
rs5945572	X	51229683	A	0.36	1.25	Genotype

Abbreviations: SNP, single nucleotide polymorphism; CHR, chromosome; POS, position; RA, risk allele; OR, odds ratio; RAF, risk allele frequency.

*RAF is based on the genome Aggregation Database (gnomAD), Non-Finnish European (NFE) population.

© 2019 Na R et al. *JAMA Network Open.*

eFigure 1. PCa Diagnosis-Free Survival Curves Based on 4-Year Follow-up of 1644 Subjects in the Placebo Arm of the REDUCE Study and Stratified by GRS Risk Groups (A), Family History (B), GRS Risk Groups in Men With a Positive Family History (C), and GRS Risk Groups in Men With a Negative Family History (D)

S1A. log-rank P<.0001

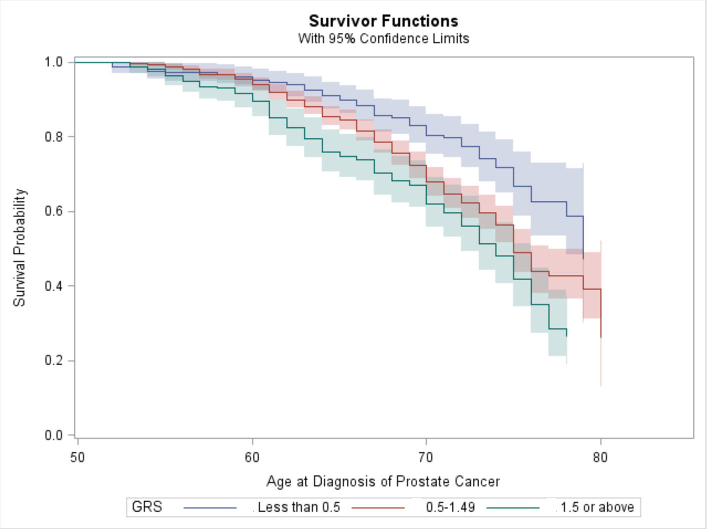

S1B. log-rank P<.0001

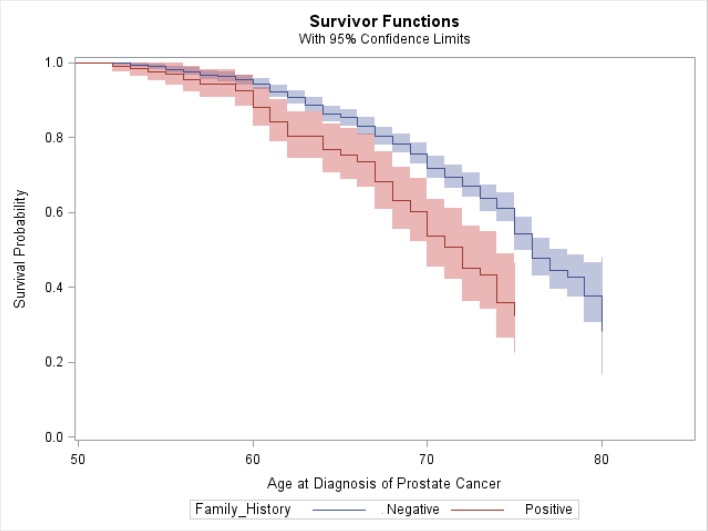

S1C. log-rank P=0.2124

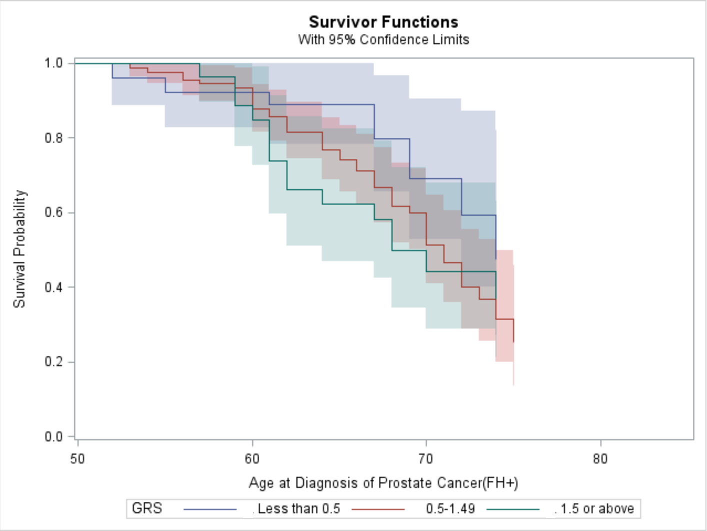

S1D. log-rank P<.0001

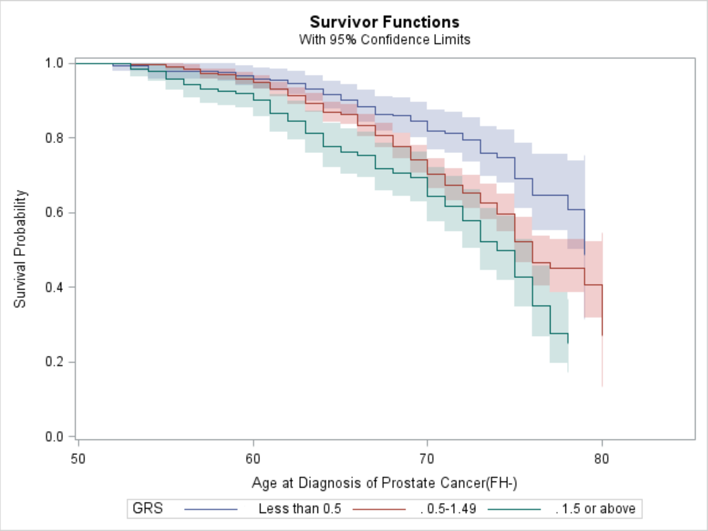

© 2019 Na R et al. *JAMA Network Open.*

eFigure 2. PCa Diagnosis-Free Survival Curves Based on 4-Year Follow-up of 1581 Subjects in the Dutasteride Arm of the REDUCE Study and Stratified by GRS Risk Groups (A), Family History (B), GRS Risk Groups in Men With a Positive Family History (C), and GRS Risk Groups in Men With a Negative Family History (D)

S2A. log-rank P<.0001

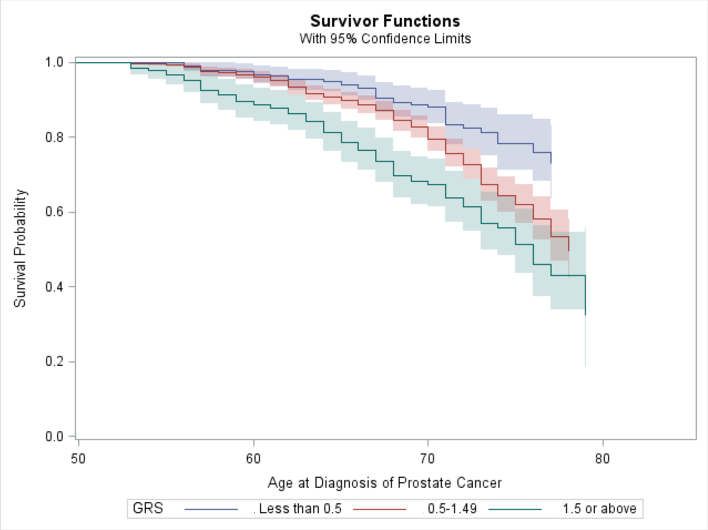

S2B. log-rank P=0.0156

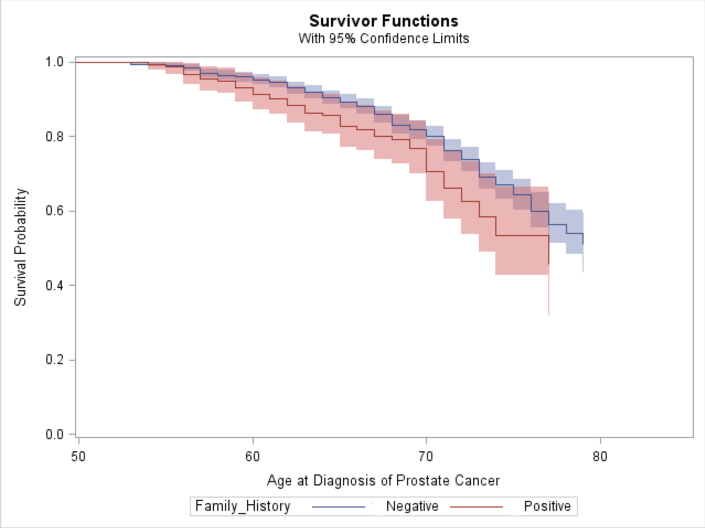

S2C. log-rank P=0.0196

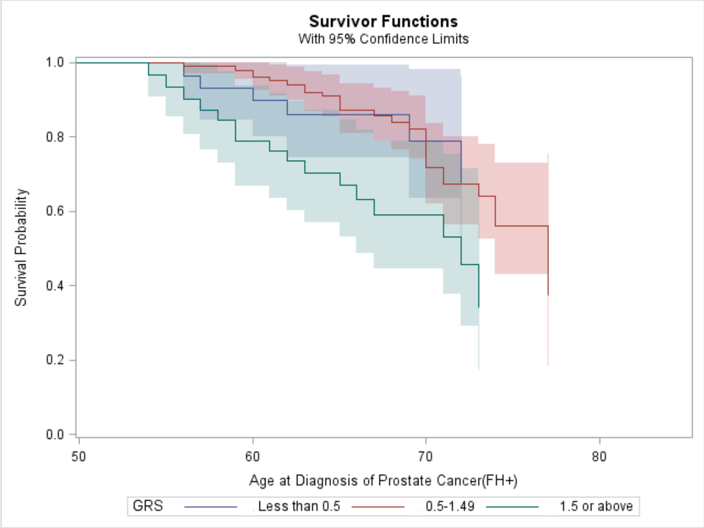

S2D. log-rank P<.0001

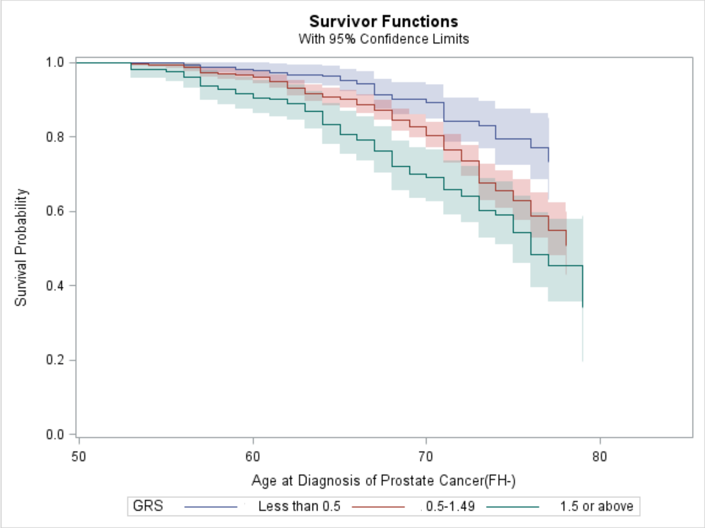

© 2019 Na R et al. *JAMA Network Open.*
